# Supplementary material for: Precision public health: A natural experiment on chronic high-contrast PM2.5 exposure and pulmonary function among older adults
Source: PLoS One. 2026 May 14;21(5):e0349025. doi: 10.1371/journal.pone.0349025 (PMC13175368; doi:10.1371/journal.pone.0349025)
Supplement: S1 File — This file contains the completed STROBE checklist, S1 Table, S2 Table, and S1 Fig. (PDF) [file pone.0349025.s001.pdf]

SUPPLEMENTARY MATERIALS

Precision Public Health Approach to Chronic $PM_{2.5}$ Exposure: A Natural Experiment on Pulmonary Function Among Older Adults Across High-Contrast Gradients

Hari Krismanuel[1*], Purnamawati Tjhin[2]

[1]Faculty of Medicine, Universitas Trisakti, Jakarta, Indonesia;

[2]Faculty of Medicine, Universitas Trisakti, Jakarta, Indonesia.

*Corresponding author: Hari Krismanuel

Email: hari_krismanuel@trisakti.ac.id

List of Contents: Page

Document S1. Completed STROBE Checklist for a cross-sectional study on the association between $PM_{2.5}$ levels and lung function among elderly. ……………………………………………………………..… 2
Table S1. Participant sociodemographic and clinical screening data for clinical vital signs, physical examination, and electrocardiogram. ……….. 4
Table S2. Participant data for anthropometrics measurements and absolute lung function parameters. ………………………………………… 8
Figure S1. Normal Q-Q Plots of Standardized Residuals for Multivariable Linear Regression Models. ………………………………………………. 13

Document S1. Completed STROBE Checklist for a cross-sectional study on the association between $PM_{2.5}$ levels and lung function among elderly.

	Item No	Recommendation	Reported on page #
**Title and abstract**	1	(a) Indicate the study's design with a commonly used term in the title or the abstract	1, 2
		(b) Provide in the abstract an informative and balanced summary of what was done and what was found	2
**Introduction**			
Background and rationale:	2	Explain the scientific background and rationale for the investigation being reported	2, 3
Objectives:	3	State specific objectives, including any prespecified hypotheses	3
**Methods**			
Study design:	4	Present key elements of study design early in the paper	3, 4
Setting:	5	Describe the setting, locations, and relevant dates, including periods of recruit-ment, data collection, and follow-up	4-12
Participants:	6	(a) Give the eligibility criteria, and the sources and methods of selection of participants	4-7
		(b) For matched studies, give matching criteria and number of exposed and unexposed	7
Variables:	7	Clearly define all outcomes, exposures, predictors, potential confounders, and effect modifiers. Give diagnostic criteria, if applicable	7-11
Data sources/measurement:	8*	For each variable of interest, give sources of data and details of methods of assessment (measurement). Describe comparability of assessment methods if there is more than one group.	7-11
Bias:	9	Describe any efforts to address potential sources of bias	8
Study size:	10	Explain how the study size was arrived at	5
Quantitative variables:	11	Explain how quantitative variables were handled in the analyses. If applicable, describe which groupings were chosen and why	11
Statistical methods:	12	(a) Describe all statistical methods, including those used to control for confounding	11
		(b) Describe any methods used to examine subgroups and interactions	11
		© Explain how missing data were addressed	11
		(d) If applicable, explain how loss to follow-up was addressed for each outcome	N/A (cross-sectional study)
		© Describe any sensitivity analyses	11
**Results**			
Participants:	13*	(a) Report numbers of individuals at each stage of study—e.g., numbers	12

		potentially eligible, examined for eligibility, confirmed eligible, included in the study, completing follow-up, and analysed<br>(b) Give reasons for non-participation at each stage	6,12
		© Consider use of a flow diagram	12
Descriptive data:	14*	(a) Give characteristics of study participants (e.g., demographic, clinical, social) and information on exposures and potential confounders	13-14
		(b) Indicate number of participants with missing data for each variable of interest	13-14 (No missing data)
Outcome data	15*	Report numbers of outcome events or summary measures	14-20
Main results	16	(a) Give unadjusted estimates and, if applicable, confounder-adjusted estimates and their precision (eg, 95% confidence interval). Make clear which confounders were adjusted for and why they were included	14
		(b) Report category boundaries when continuous variables were categorized	14-16
		© If relevant, consider translating estimates of relative risk into absolute risk for a meaningful time period	N/A
Other analyses	17	Report other analyses done—eg analyses of subgroups and interactions, and sensitivity analyses	11, 14-20
**Discussion**			
Key results	18	Summarise key results with reference to study objectives	20-21
Limitations	19	Discuss limitations of the study, taking into account sources of potential bias or imprecision. Discuss both direction and magnitude of any potential bias	21-22
Interpretation	20	Give a cautious overall interpretation of results considering objectives, limitations, multiplicity of analyses, results from similar studies, and other relevant evidence	20-22
Generalisability	21	Discuss the generalisability (external validity) of the study results	22
**Other information**			
Funding	22	Give the source of funding and the role of the funders for the present study and, if applicable, for the original study on which the present article is based	N/A

*Give information separately for exposed and unexposed groups.

Table S1. Participant sociodemographic and clinical screening data for clinical vital signs, physical examination, and electrocardiogram

ID	Group	Gender	Ethnic	Age	Education level	Occupa-tion	SBP	DBP	HR	RR	Temp. (ºC)	SpO_2 (%)	Heart & lungs	ECG
1	1	2	4	73	2	1	123	75	80	20	36,5	96	WNL	sinus rhythm
3	1	2	4	70	1	1	121	80	76	19	36,8	96	WNL	sinus rhythm
5	1	2	4	72	1	1	125	78	76	19	36,2	96	WNL	sinus rhythm
6	1	2	4	71	1	1	120	78	78	20	36,1	97	WNL	sinus rhythm
7	1	2	4	70	1	1	122	75	79	19	36,1	96	WNL	sinus rhythm
8	1	2	4	74	2	1	122	80	74	18	36,6	96	WNL	sinus rhythm
10	1	2	4	73	2	1	117	71	71	19	36,2	95	WNL	sinus rhythm
12	1	2	4	69	1	1	126	80	71	18	36,4	96	WNL	sinus rhythm
13	1	2	4	72	1	1	125	77	82	18	36,6	97	WNL	sinus rhythm
14	1	1	4	67	1	1	127	72	63	20	36,2	97	WNL	sinus rhythm
15	1	1	4	73	1	1	127	70	78	19	36,4	96	WNL	sinus rhythm
17	1	1	4	70	1	1	120	79	82	19	37,5	96	WNL	sinus rhythm
18	1	2	4	69	1	1	126	80	77	20	36,6	97	WNL	sinus rhythm
19	1	1	4	72	1	1	122	74	78	19	36,1	95	WNL	sinus rhythm
20	1	2	4	72	1	1	126	79	82	20	36,0	96	WNL	sinus rhythm
21	1	2	4	75	1	1	125	79	71	19	36,4	97	WNL	sinus rhythm
22	1	2	4	80	1	1	122	71	78	20	36,6	95	WNL	sinus rhythm
23	1	1	4	70	1	1	123	67	66	20	36,0	95	WNL	sinus rhythm
24	1	2	4	72	1	1	123	75	73	19	36,8	97	WNL	sinus rhythm
25	1	2	4	67	1	1	123	72	75	19	36,3	97	WNL	sinus rhythm
27	1	2	4	73	1	1	119	78	79	20	36,1	95	WNL	sinus rhythm
28	1	2	4	69	2	1	113	71	85	18	36,6	96	WNL	sinus rhythm
30	1	2	4	75	1	1	115	69	76	19	36,1	96	WNL	sinus rhythm
31	1	2	4	66	1	1	117	70	88	18	36,0	97	WNL	sinus rhythm
32	1	2	4	74	1	1	123	71	87	18	36,4	95	WNL	sinus rhythm
34	1	2	4	72	1	1	117	71	76	20	37,3	95	WNL	sinus rhythm
35	1	2	4	71	1	1	119	76	80	20	36,4	97	WNL	sinus rhythm
36	1	2	4	68	2	1	110	75	73	19	36,3	96	WNL	sinus rhythm
37	1	1	4	69	2	1	125	72	72	20	36,6	96	WNL	sinus rhythm
38	1	2	4	74	1	1	112	75	79	19	35,8	96	WNL	sinus rhythm

39	1	1	4	68	1	1	119	75	71	19	36,7	97	WNL	sinus rhythm
40	1	2	4	72	1	1	117	76	81	19	36,4	95	WNL	sinus rhythm
41	1	2	4	70	1	1	122	71	76	18	36,3	96	WNL	sinus rhythm
42	1	2	4	74	1	1	119	76	88	20	36,8	95	WNL	sinus rhythm
43	1	1	4	68	1	1	123	73	73	18	36,8	97	WNL	sinus rhythm
49	1	2	4	73	1	1	119	75	82	20	36,4	95	WNL	sinus rhythm
50	1	2	4	72	1	1	122	76	86	19	36,6	95	WNL	sinus rhythm
51	1	2	4	71	3	1	118	74	78	18	36,4	96	WNL	sinus rhythm
52	1	2	4	70	2	1	123	79	77	19	36,8	96	WNL	sinus rhythm
53	1	2	4	73	1	1	120	78	76	19	36,3	96	WNL	sinus rhythm
54	1	2	4	73	1	1	119	78	89	19	36,8	97	WNL	sinus rhythm
55	1	1	4	70	2	1	115	74	84	19	36,7	96	WNL	sinus rhythm
56	1	2	4	75	1	1	117	72	77	19	36,5	95	WNL	sinus rhythm
57	1	2	4	71	1	1	121	70	82	20	36,2	96	WNL	sinus rhythm
58	1	1	4	69	2	1	124	75	77	18	36,7	96	WNL	sinus rhythm
59	1	2	4	70	1	1	118	70	77	20	36,0	95	WNL	sinus rhythm
60	1	2	4	71	1	1	120	76	76	20	36,1	96	WNL	sinus rhythm
1	2	1	4	78	1	1	123	75	72	18	36,4	95	WNL	sinus rhythm
3	2	2	4	75	1	1	118	68	73	18	36,2	98	WNL	sinus rhythm
4	2	2	4	70	2	1	124	71	80	19	36,2	96	WNL	sinus rhythm
5	2	2	4	75	1	1	114	71	84	19	37,0	95	WNL	sinus rhythm
6	2	2	4	72	1	1	112	77	80	18	36,8	96	WNL	sinus rhythm
7	2	2	4	73	1	1	112	78	76	18	36,0	96	WNL	sinus rhythm
8	2	2	4	68	1	1	118	73	88	20	36,6	96	WNL	sinus rhythm
10	2	2	4	80	1	1	124	79	87	20	36,0	97	WNL	sinus rhythm
11	2	2	4	72	1	1	119	73	82	20	36,8	95	WNL	sinus rhythm
12	2	2	4	70	1	1	118	69	87	17	36,8	95	WNL	sinus rhythm
15	2	2	4	73	1	1	119	75	81	19	36,1	95	WNL	sinus rhythm
16	2	2	4	72	1	1	118	76	96	18	36,9	96	WNL	sinus rhythm
17	2	2	4	69	2	1	118	72	81	19	36,1	96	WNL	sinus rhythm
18	2	2	4	75	3	1	117	68	61	18	36,1	97	WNL	sinus rhythm
19	2	2	4	68	1	1	113	67	81	19	36,2	96	WNL	sinus rhythm
20	2	2	4	66	1	1	124	75	70	18	36,1	96	WNL	sinus rhythm
21	2	2	4	70	1	1	127	75	85	19	36,6	96	WNL	sinus rhythm

22	2	2	4	70	1	1	117	70	71	17	36,7	96	WNL	sinus rhythm
23	2	2	4	72	1	1	118	70	81	18	36,6	97	WNL	sinus rhythm
24	2	2	4	79	1	1	118	71	82	18	36,6	96	WNL	sinus rhythm
25	2	2	4	69	1	1	111	76	77	19	36,4	96	WNL	sinus rhythm
27	2	1	4	72	2	1	124	77	81	19	36,2	97	WNL	sinus rhythm
29	2	1	4	79	2	1	123	71	71	19	36,7	96	WNL	sinus rhythm
30	2	2	4	68	1	1	119	76	77	19	36,2	96	WNL	sinus rhythm
31	2	2	4	74	2	1	117	78	83	18	35,9	97	WNL	sinus rhythm
32	2	2	4	73	2	1	118	80	75	19	35,8	96	WNL	sinus rhythm
33	2	2	4	70	1	1	113	67	69	18	35,6	96	WNL	sinus rhythm
34	2	2	4	74	1	1	111	71	78	18	36,1	96	WNL	sinus rhythm
35	2	2	4	67	1	1	119	79	74	19	36,3	96	WNL	sinus rhythm
36	2	2	4	74	1	1	108	71	88	18	36,3	96	WNL	sinus rhythm
37	2	2	4	67	2	1	114	74	83	19	36,4	96	WNL	sinus rhythm
41	2	1	4	75	2	1	126	67	84	20	36,2	97	WNL	sinus rhythm
42	2	2	4	69	1	1	120	78	88	20	36,1	97	WNL	sinus rhythm
43	2	2	4	73	1	1	123	73	88	20	36,3	97	WNL	sinus rhythm
44	2	2	4	69	1	1	122	73	79	20	36,4	97	WNL	sinus rhythm
45	2	2	4	69	2	1	116	77	71	18	36,5	96	WNL	sinus rhythm
46	2	2	4	78	2	1	126	73	83	20	36,7	97	WNL	sinus rhythm
47	2	2	4	66	2	1	126	73	78	18	36,3	96	WNL	sinus rhythm
48	2	2	4	69	1	1	118	80	68	18	36,2	96	WNL	sinus rhythm
50	2	2	4	65	2	1	118	71	82	19	36,4	96	WNL	sinus rhythm
51	2	2	4	68	2	1	119	72	83	19	36,5	95	WNL	sinus rhythm
53	2	2	4	65	3	1	117	73	84	19	36,5	96	WNL	sinus rhythm
54	2	2	4	72	1	1	120	71	82	19	36,4	95	WNL	sinus rhythm
55	2	2	4	70	1	1	116	76	74	18	36,4	95	WNL	sinus rhythm
56	2	1	4	73	1	1	119	76	67	20	36,8	95	WNL	sinus rhythm
60	2	2	4	72	1	1	118	70	77	19	36,2	97	WNL	sinus rhythm
61	2	2	4	72	1	1	124	78	72	19	36,9	96	WNL	sinus rhythm
62	2	2	4	72	1	1	122	75	82	20	36,3	97	WNL	sinus rhythm
63	2	2	4	74	1	1	127	71	71	18	36,2	95	WNL	sinus rhythm
64	2	1	4	74	1	1	110	72	73	20	36,0	95	WNL	sinus rhythm
65	2	2	4	73	1	1	124	71	70	19	36,2	96	WNL	sinus rhythm

66	2	1	4	67	1	1	122	70	81	20	36,2	95	WNL	sinus rhythm
67	2	2	4	73	2	1	126	73	76	18	36,3	95	WNL	sinus rhythm
68	2	2	4	70	1	1	120	73	61	17	36,5	95	WNL	sinus rhythm

Notes:
Gender: 1 = Male, 2 = Female.
Group: 1 = Low $PM_{2.5}$ exposure, 2 = High $PM_{2.5}$ exposure.
Education: Categorized into three levels: 1 = Low, 2 = Middle, 3 = High.
Occupation: Categorized as: 1 = Unemployed/informal, 2 = Employee, 3 = Professional.
SBP: Systolic Blood Pressure (mmHg); DBP: Diastolic Blood Pressure (mmHg); HR: Heart Rate (beats per minute); RR: Respiratory Rate (breaths per minute); Temp: Temperature (°C); SpO2: Peripheral Oxygen Saturation (%); WNL: Within Normal Limits; ECG: Electrocardiogram.

Table S2. Participant data for anthropometrics measurements and absolute lung function parameters.

No.	Group	Sex	Gender	Ethnic	Age	Height	BMI	FEV_1_actual	FVC_actual	FEV_1/FVC_actual	Gender_Text	Ethnic_Text
1	1	F	2	4	73	1,49	18,74	1,21	1,68	0,72	Female	SoutheastAsian
3	1	F	2	4	70	1,44	20,35	1,12	1,24	0,90	Female	SoutheastAsian
5	1	F	2	4	72	1,45	20,98	1,84	1,90	0,93	Female	SoutheastAsian
6	1	F	2	4	71	1,3	22,19	1,70	1,72	0,97	Female	SoutheastAsian
7	1	F	2	4	70	1,4	20,77	1,35	1,55	0,87	Female	SoutheastAsian
8	1	F	2	4	74	1,45	21,21	1,76	1,80	0,76	Female	SoutheastAsian
10	1	F	2	4	73	1,48	21,00	1,78	1,91	0,86	Female	SoutheastAsian
12	1	F	2	4	69	1,49	20,40	1,02	1,46	0,70	Female	SoutheastAsian
13	1	F	2	4	72	1,5	21,33	1,05	1,31	0,80	Female	SoutheastAsian
14	1	M	1	4	67	1,61	20,52	1,64	1,87	0,88	Male	SoutheastAsian
15	1	M	1	4	73	1,61	20,25	1,78	1,85	0,92	Male	SoutheastAsian
17	1	M	1	4	70	1,55	20,15	1,63	1,83	0,89	Male	SoutheastAsian
18	1	F	2	4	69	1,5	21,78	1,90	1,98	0,92	Female	SoutheastAsian
19	1	M	1	4	72	1,54	19,40	0,88	1,05	0,74	Male	SoutheastAsian
20	1	F	2	4	72	1,54	21,08	1,74	1,75	0,99	Female	SoutheastAsian
21	1	F	2	4	75	1,45	20,93	1,05	1,21	0,87	Female	SoutheastAsian
22	1	F	2	4	80	1,35	19,75	1,29	1,47	0,91	Female	SoutheastAsian
23	1	M	1	4	70	1,5	20,44	2,54	3,00	0,85	Male	SoutheastAsian
24	1	F	2	4	72	1,44	21,70	1,54	1,56	0,99	Female	SoutheastAsian
25	1	F	2	4	67	1,44	22,18	2,24	2,25	1,00	Female	SoutheastAsian

27	1	F	2	4	73	1,5	20,00	1,42	1,78	0,80	Female	SoutheastAsian
28	1	F	2	4	69	1,55	20,40	2,74	2,77	0,99	Female	SoutheastAsian
30	1	F	2	4	75	1,35	20,30	1,58	1,86	0,85	Female	SoutheastAsian
31	1	F	2	4	66	1,6	21,48	1,58	1,62	0,98	Female	SoutheastAsian
32	1	F	2	4	74	1,4	20,92	1,87	1,88	0,99	Female	SoutheastAsian
34	1	F	2	4	72	1,4	21,43	1,90	1,93	0,98	Female	SoutheastAsian
35	1	F	2	4	71	1,48	20,77	1,91	1,92	0,99	Female	SoutheastAsian
36	1	F	2	4	68	1,45	20,21	1,00	1,16	0,86	Female	SoutheastAsian
37	1	M	1	4	69	1,54	21,29	2,10	2,17	0,97	Male	SoutheastAsian
38	1	F	2	4	74	1,4	19,18	0,92	0,96	0,96	Female	SoutheastAsian
39	1	M	1	4	68	1,45	21,02	1,22	1,22	1,00	Male	SoutheastAsian
40	1	F	2	4	72	1,47	19,44	1,05	1,14	0,92	Female	SoutheastAsian
41	1	F	2	4	70	1,5	20,89	1,38	1,38	1,00	Female	SoutheastAsian
42	1	F	2	4	74	1,44	21,70	1,85	1,86	0,99	Female	SoutheastAsian
43	1	M	1	4	68	1,5	20,89	1,40	1,40	1,00	Male	SoutheastAsian
49	1	F	2	4	73	1,45	20,93	0,88	0,91	0,97	Female	SoutheastAsian
50	1	F	2	4	72	1,44	20,74	1,03	1,19	0,87	Female	SoutheastAsian
51	1	F	2	4	71	1,54	21,50	1,04	1,04	1,00	Female	SoutheastAsian
52	1	F	2	4	70	1,45	20,93	1,26	1,39	0,91	Female	SoutheastAsian
53	1	F	2	4	73	1,5	20,89	1,05	1,06	0,99	Female	SoutheastAsian
54	1	F	2	4	73	1,4	20,41	0,97	1,05	0,92	Female	SoutheastAsian
55	1	M	1	4	70	1,6	21,09	1,86	1,88	0,99	Male	SoutheastAsian
56	1	F	2	4	75	1,55	20,40	1,44	1,47	0,98	Female	SoutheastAsian
57	1	F	2	4	71	1,35	19,20	1,66	1,79	0,84	Female	SoutheastAsian

58	1	M	1	4	69	1,6	19,92	1,48	1,49	0,99	Male	SoutheastAsian
59	1	F	2	4	70	1,45	20,45	1,90	1,90	1,00	Female	SoutheastAsian
60	1	F	2	4	71	1,45	20,93	1,03	1,07	0,96	Female	SoutheastAsian
1	2	M	1	4	78	154	21,08	1,18	1,48	0,80	Male	SoutheastAsian
3	2	F	2	4	75	142	20,78	0,85	1,03	0,83	Female	SoutheastAsian
4	2	F	2	4	70	155	20,94	2,00	2,40	0,83	Female	SoutheastAsian
5	2	F	2	4	75	151	20,17	0,35	0,35	1,00	Female	SoutheastAsian
6	2	F	2	4	72	149	20,67	1,04	1,19	0,87	Female	SoutheastAsian
7	2	F	2	4	73	151	21,71	0,69	0,88	0,78	Female	SoutheastAsian
8	2	F	2	4	68	157	20,73	1,03	1,26	0,82	Female	SoutheastAsian
10	2	F	2	4	80	148	19,81	1,33	1,33	1,00	Female	SoutheastAsian
11	2	F	2	4	72	151	20,88	1,20	1,20	1,00	Female	SoutheastAsian
12	2	F	2	4	70	147	18,74	1,16	1,43	0,81	Female	SoutheastAsian
15	2	F	2	4	73	153	21,79	1,37	1,39	0,99	Female	SoutheastAsian
16	2	F	2	4	72	149	20,99	0,89	0,91	0,98	Female	SoutheastAsian
17	2	F	2	4	69	146	21,86	1,00	1,04	0,96	Female	SoutheastAsian
18	2	F	2	4	75	142	20,48	0,74	0,79	0,94	Female	SoutheastAsian
19	2	F	2	4	68	144	20,40	0,92	1,10	0,84	Female	SoutheastAsian
20	2	F	2	4	66	154	21,29	1,51	1,90	0,79	Female	SoutheastAsian
21	2	F	2	4	70	148	20,77	1,16	1,32	0,88	Female	SoutheastAsian
22	2	F	2	4	70	146	20,97	0,73	0,78	0,94	Female	SoutheastAsian
23	2	F	2	4	72	145	20,93	0,71	0,71	1,00	Female	SoutheastAsian
24	2	F	2	4	79	160	20,08	0,58	0,65	0,89	Female	SoutheastAsian
25	2	F	2	4	69	156	19,60	1,52	1,77	0,86	Female	SoutheastAsian

27	2	M	1	4	72	157	19,35	1,73	1,75	0,99	Male	SoutheastAsian
29	2	M	1	4	79	162	22,14	1,63	1,83	0,89	Male	SoutheastAsian
30	2	F	2	4	68	140	20,97	1,48	1,55	0,95	Female	SoutheastAsian
31	2	F	2	4	74	148	20,82	1,18	1,27	0,93	Female	SoutheastAsian
32	2	F	2	4	73	144	20,93	0,74	0,76	0,97	Female	SoutheastAsian
33	2	F	2	4	70	149	21,26	0,86	1,14	0,75	Female	SoutheastAsian
34	2	F	2	4	74	153	21,32	0,64	0,64	1,00	Female	SoutheastAsian
35	2	F	2	4	67	153	21,23	1,48	2,15	0,69	Female	SoutheastAsian
36	2	F	2	4	74	148	20,45	0,94	0,94	1,00	Female	SoutheastAsian
37	2	F	2	4	67	144	20,40	1,74	1,77	0,98	Female	SoutheastAsian
41	2	M	1	4	75	155	19,15	1,50	1,94	0,77	Male	SoutheastAsian
42	2	F	2	4	69	151	20,61	1,16	1,16	1,00	Female	SoutheastAsian
43	2	F	2	4	73	139	20,13	0,76	1,29	0,59	Female	SoutheastAsian
44	2	F	2	4	69	160	20,70	0,74	0,74	1,00	Female	SoutheastAsian
45	2	F	2	4	69	156	20,92	1,34	1,34	1,00	Female	SoutheastAsian
46	2	F	2	4	78	146	20,69	1,05	1,06	0,99	Female	SoutheastAsian
47	2	F	2	4	66	147	20,41	1,50	1,5	1,00	Female	SoutheastAsian
48	2	F	2	4	69	153	21,40	0,95	0,95	1,00	Female	SoutheastAsian
50	2	F	2	4	65	156	21,00	1,96	2,03	0,97	Female	SoutheastAsian
51	2	F	2	4	68	155	21,27	1,78	1,89	0,94	Female	SoutheastAsian
53	2	F	2	4	65	151	21,71	1,78	1,84	0,97	Female	SoutheastAsian
54	2	F	2	4	72	151	21,05	0,66	0,66	1,00	Female	SoutheastAsian
55	2	F	2	4	70	154	20,87	1,54	1,78	0,87	Female	SoutheastAsian
56	2	M	1	4	73	162	22,48	1,26	1,56	0,81	Male	SoutheastAsian

60	2	F	2	4	72	145	20,74	1,11	1,55	0,72	Female	SoutheastAsian
61	2	F	2	4	72	145	21,64	0,76	0,79	0,96	Female	SoutheastAsian
62	2	F	2	4	72	147	21,15	0,96	1,27	0,76	Female	SoutheastAsian
63	2	F	2	4	74	154	20,96	0,87	0,93	0,94	Female	SoutheastAsian
64	2	M	1	4	74	164	19,93	0,87	1,31	0,66	Male	SoutheastAsian
65	2	F	2	4	73	153	20,16	1,15	1,29	0,89	Female	SoutheastAsian
66	2	M	1	4	67	160	20,70	2,59	2,60	1,00	Male	SoutheastAsian
67	2	F	2	4	73	163	20,96	1,55	1,65	0,94	Female	SoutheastAsian
68	2	F	2	4	70	157	21,18	1,25	1,45	0,86	Female	SoutheastAsian

Notes:

- **Group:** Classification of participants based on residential location and air pollution exposure.
  - **1:** Low-Exposure Group.
  - **2:** High-Exposure Group.
- **Sex/Gender:**
  - **M / 1:** Male.
  - **F / 2:** Female.
- **Ethnic/Ethnic_Text:** All participants were classified as **"SoutheastAsian"** (Code 4) based on the Global Lung Function Initiative (GLI-2012) ethnicity reference to ensure accurate z-score calculations.
- **Height:** Measured in meters (m).
- **Age:** Reported in years.
- **Spirometric Parameters:**
  - **FEV_1_actual:** Forced Expiratory Volume in 1 second, measured in Liters (L).
  - **FVC_actual:** Forced Vital Capacity, measured in Liters (L).
  - **FEV_1/FVC_actual:** The absolute ratio of FEV1 to FVC.
- **Data Integrity:** All 101 participants listed above passed the initial clinical screening, including vital signs within normal limits and absence of acute respiratory symptoms at the time of testing.
- **Z-score Reproducibility**: The spirometric z-scores (FEV1_z, FVC_z, FEV1/FVC_z) used in the regression analyses can be reproduced from the absolute spirometric values provided above using the Global Lung Function Initiative (GLI-2012) reference equations for the SoutheastAsian ethnicity (Code 4), based on age, sex, and height variables included in this dataset.

Figure S1. Normal Q-Q Plots of Standardized Residuals for Multivariable Linear Regression Models.

Figure S1A. Normal Q-Q plot of residuals for the FEV_1 z-score model.

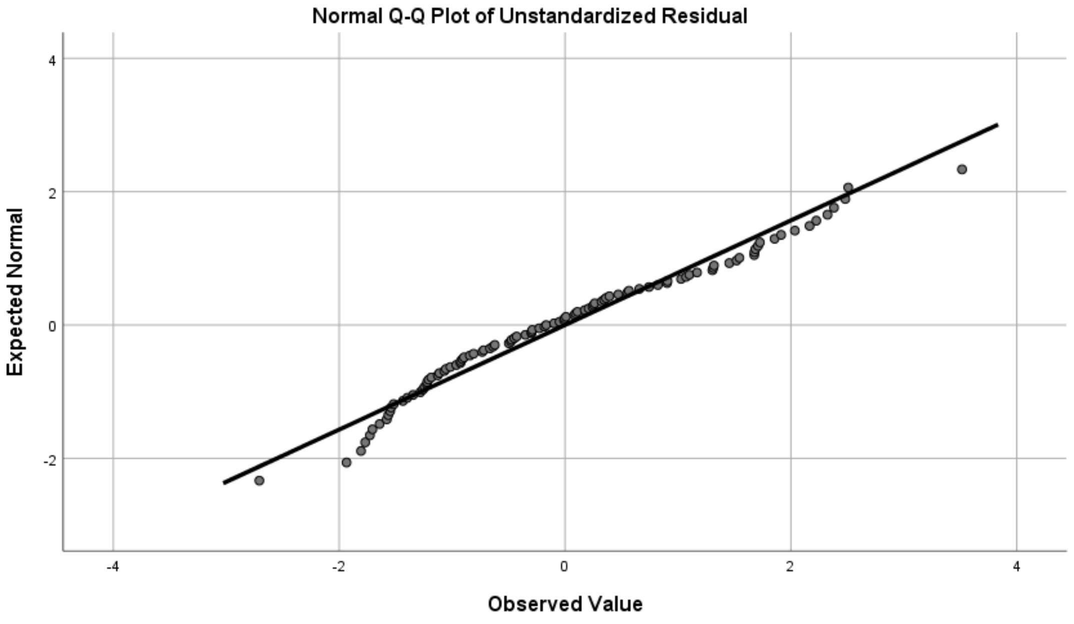

Figure S1B. Normal Q-Q plot of residuals for the FVC z-score model.

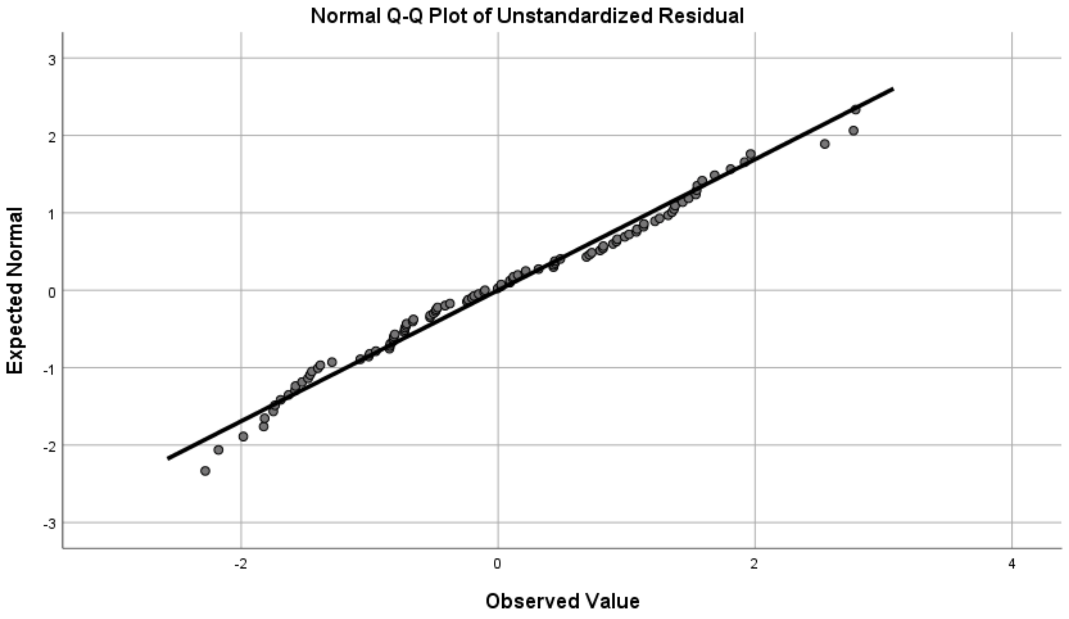

Figure S1C. Normal Q-Q plot of residuals for the FEV_1/ FVC z-score model.

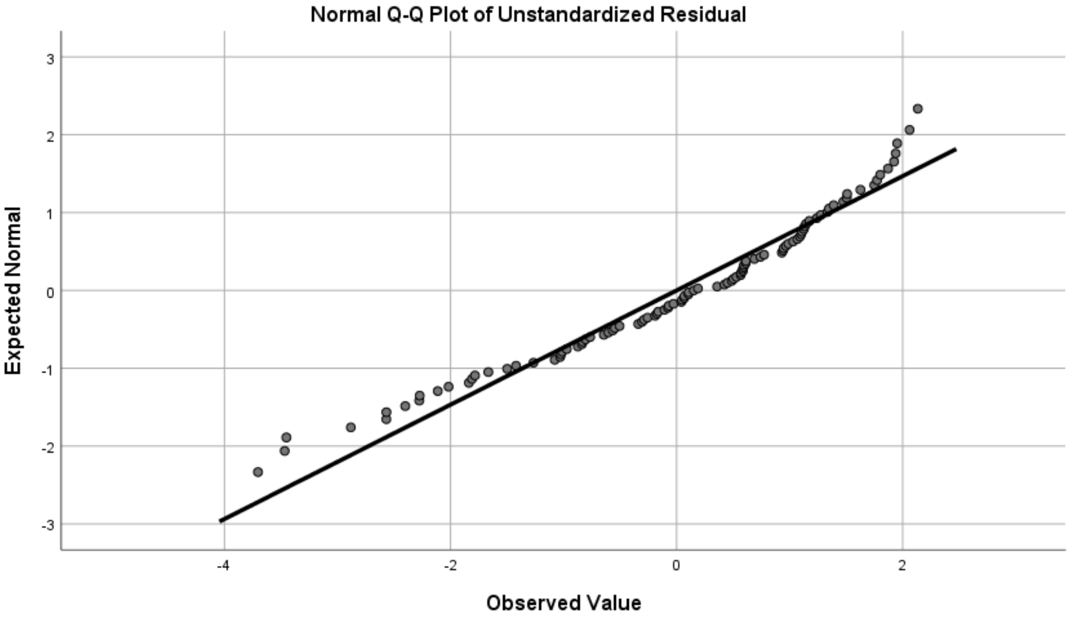

Figure S1. Normal Q-Q Plots of Standardized Residuals for Multivariable Linear Regression Models.
The plots demonstrate that the standardized residuals of all three regression models (FEV_1_z, FVC_z, and FEV_1 /FVCz) follow an approximately linear pattern along the 45-degree reference line. While minor departures are noted at the extremes, the absence of substantial skewness or systematic bowing supports the assumption of approximate normality. This visual evidence, combined with the moderate sample size (n=101), validates the robustness of the parametric estimates and confirms that the regression models are not biased by non-normal error distributions.
