# Supplementary material for: Precision public health: A natural experiment on chronic high-contrast PM2.5 exposure and pulmonary function among older adults
Source: PLoS One. 2026 May 14;21(5):e0349025. doi: 10.1371/journal.pone.0349025 (PMC13175368; doi:10.1371/journal.pone.0349025)
Supplement: Attachment — Submitted filename: Response to Academic Editor and Reviewers.docx [file pone.0349025.s003.docx]

**Response to Academic Editor**

**Manuscript ID: PONE-D-26-04311
Title: Precision public health: a natural experiment on chronic high-contrast PM2.5 exposure and pulmonary function among older adults**

**Dear Dr. George Kuryan,
Academic Editor, PLOS ONE,**

**Thank you for the opportunity to revise our manuscript. We sincerely appreciate the constructive guidance provided by you and the reviewers. Below, we address the specific journal requirements:**

**1.	Style Requirements and File Naming**

**Response: We have carefully reviewed the PLOS ONE formatting guidelines and templates. The revised manuscript has been reformatted to comply fully with the journal’s style requirements, including title page structure, author affiliations, section organization, heading hierarchy, and main body formatting. All tables and figures have been prepared according to PLOS ONE specifications, and all submission files have been named in accordance with the journal’s file naming conventions.**

**2.	Data Availability Statement**

**Response: We confirm that the submission includes the full de-identified participant-level dataset constituting the minimal data set required to replicate all findings reported in the manuscript. The Supporting Information files contain all variables used in the statistical analyses, including exposure group, sociodemographic characteristics, anthropometric measurements, and absolute spirometric parameters. These data provide the values underlying all reported means, standard deviations, regression analyses, and figures.
There are no ethical or legal restrictions on sharing the de-identified dataset.**

**3.	Recommendations to Cite Specific Works**

**Response: The reviewers did not request citation of specific additional publications. We have nevertheless carefully reviewed the reference list to ensure that all references are appropriate, relevant, and up to date.**

**We have also addressed all comments from Reviewer #1 and Reviewer #2 in the accompanying 'Response to Reviewers' document.**

**Thank you for your continued consideration of our work.**

**Sincerely,**

**Dr. Hari Krismanuel**

**Response to Reviewers (Rebuttal)**

**Response to Reviewers' General Assessments**

**1.	Technical Soundness and Support for Conclusions**

- **Reviewer #1: Partly**
- **Reviewer #2: Yes**

**Response: We thank Reviewer #2 for their positive evaluation of the study’s technical soundness. We also appreciate Reviewer #1’s assessment and have carefully reviewed the manuscript to ensure that all conclusions are fully supported by the data presented.**

**In response, we have:**

- **Moderated causal language to ensure conclusions remain consistent with the observational design**
- **Clarified that associations are interpreted within the constraints of area-level exposure assignment**
- **Refined the spirometric interpretation to avoid overdiagnosis (i.e., “pattern consistent with reduced lung volumes”)**
- **Strengthened statistical justification and model diagnostics reporting**

**These revisions ensure that the conclusions are proportionate to the data presented, methodologically justified, and fully supported by the analytical framework.**

**2.	Has the statistical analysis been performed appropriately and rigorously?**

- **Reviewer #1: No**
- **Reviewer #2: Yes**

**Response:** We acknowledge Reviewer #1’s concerns regarding the statistical presentation and have implemented the following improvements to ensure methodological rigor:

- **Normality & Robustness: We added a Q-Q plot and conducted a sensitivity analysis (Cook’s Distance < 0.15), confirming that no influential outliers affected the regression estimates despite slight deviations in formal normality tests.**
- **Clinical Interpretation of Effect Sizes: We have expanded the discussion on effect sizes, specifically interpreting the large Cohen’s d (1.21 for FEV1_z, and 1.02 for FVC_z). We now explicitly state that a reduction of more than 1 SD corresponds to a significant percentile shift in lung function (e.g., from the 50th to the 15th percentile), highlighting its clinical magnitude.**
- **Variance Explanation (R^2^): We have highlighted the strong R^2^ values (31.5% for FEV_1__z and 25.9% for FVC_z) to emphasize that PM2.5 exposure explains a substantial proportion of the variance in pulmonary function relative to other demographic variables.**
- **Power Calculation: To further clarify the adequacy of our sample size, we provided an achieved power estimation based on the observed effect sizes. With n=101 and the observed large effect sizes, the study achieved a statistical power of >0.95 at α=0.01, confirming the study was more than adequately powered to detect the reported differences.**

**3.	Data Availability**

- **Reviewer #1: Yes**
- **Reviewer #2: Yes**

**Response:** We are pleased that both reviewers found our data availability to be transparent and compliant with the journal's requirements. We confirm that all relevant data underlying the findings of this study are available within the manuscript and its **Supporting Information** files. Specifically, we have ensured the following:

- **The minimal data set required to replicate all study findings, including individual data points behind the means, standard deviations, and measures reported, is included.**
- The data used to build all graphs and tables presented in the manuscript are fully available as **Supporting Information**.
- There are no ethical or legal restrictions on sharing the de-identified data set.

**4.	Manuscript Presentation and English Language**

- **Reviewer #1: Yes**
- **Reviewer #2: Yes**

**Response:** We thank both reviewers for their positive assessment of the clarity and quality of our English writing. To ensure that the manuscript remains clear, correct, and unambiguous as per PLOS ONE’s standards:

- We have conducted a final thorough proofreading of the entire manuscript to eliminate any remaining minor typographical or grammatical errors.
- We have ensured that all technical and physiological terms are used consistently and correctly throughout the revised text.

**5.	Response to Reviewer #1 Comments**

**We sincerely thank Reviewer #1 for the thorough, constructive critique** and **highly insightful evaluation** of our manuscript**. We appreciate the recognition of our methodological strengths, including the 10-year residency filter, the use of GLI-2012 z-scores,** and **the study’s translational relevance. Below, we provide a detailed, point-by-point response to all comments and describe the revisions made in the revised manuscript. All major concerns have been carefully addressed as outlined below.**

**1.	Overstatement of “Global First”**

- **Reviewer Comment: Claims of "first globally" and "pioneering application globally" are overstated.**
- **Response: We appreciate this important observation and agree that our original wording may have overstated the novelty of the study. While the integration of a decade-long residency filter, high-contrast exposure setting, and GLI-2012 SEA z-scores is relatively uncommon, we acknowledge that similar methodological elements have been used in other contexts.**
- **Revision: We have removed all “global first” and “pioneering” claims throughout the manuscript. The text has been reframed to: *"To our knowledge, this study is among the first in the Southeast Asian context to utilize a decade-long stable residency filter..."* (See page 1, lines 30-35).**

**2.	Causal Language and RCT Comparisons**

- **Reviewer Comment: Causal language exceeds the evidentiary strength; design is not equivalent to RCT.**
- **Response: We agree that as an observational study, we must be more conservative. We have toned down the causal language. We now explicitly state that while the 10-year residency filter strengthens temporal plausibility and internal validity, the study strengthens causal inference within observational constraints but does not establish definitive causality.**
- **Revision:**
  - **Changed "primary driver" to *"strong independent association consistent with a chronic exposure effect."***
  - **Changed "approaches causal rigor of RCT" to *"strengthens causal inference within the inherent constraints of an observational design."***

**3.	Area-Level Exposure Proxy Limitations**

- **Reviewer Comment: Limitation of binary exposure (Kedoya vs. Pangalengan) and lack of personal monitoring are underplayed.**
- **Response: We agree and thank the reviewer for highlighting this important issue. While the high-contrast natural experiment design provides strong exposure differentiation, we acknowledge limitations inherent to ecological exposure assignment.** **We have revised the Limitations section to be more transparent and less defensive.**
- **Revision: The manuscript now explicitly acknowledges the following limitations: (1) Potential risk of ecological exposure assignment; (2) Uncaptured within-area variability; (3) Absence of personal/indoor exposure monitoring; (4) Lack of cumulative dose quantification; (5) Absence of time–activity pattern analysis; and (6) Absence of historical exposure trajectory modeling.**

**These clarifications have been added to the Limitations section (Please see Revised Manuscript, page 23, lines 736–748).**

**4.	Restrictive Pattern Interpretation**

- **Reviewer Comment: Spirometry alone cannot diagnose "restriction"; it shows a "restrictive pattern."**
- **Response: We appreciate this physiological nuance. We have corrected the terminology to avoid any definitive implication of parenchymal stiffening, acknowledging that spirometry measures airflow and volumes rather than tissue compliance.**
- **Revision: We have replaced "restrictive-type impairment" with "restrictive-pattern spirometry" or "pattern consistent with reduced lung volumes" throughout the manuscript, including the Abstract, Results, and Discussion sections. (Please see Revised Manuscript, page 1, lines 45-47)**

**5.	Statistical Justification (Normality and Effect Sizes)**

- **Reviewer Comment: S-W for needs better justification. Also, clinical magnitude of Cohen’s should be interpreted.**
- **Response: We appreciate this important statistical observation. We have significantly strengthened the reporting of model diagnostics and clarified the robustness of our parametric estimates. Although the Shapiro–Wilk test indicated deviation from strict normality in the FEV₁/FVC_z model (p = 0.001), visual inspection of residual Q–Q plots demonstrated approximate normal distribution without substantial skewness or kurtosis. Moreover, given the moderate sample size (n = 101), linear regression estimates are generally robust to minor deviations from normality. We further clarify that the normality assumption in linear regression pertains to the distribution of model residuals rather than the raw outcome variables. Therefore, residual diagnostics such as Q–Q plots and influence statistics provide the appropriate basis for evaluating model validity. We have also expanded the interpretation of effect size magnitude to clarify clinical relevance. In addition, we clarified in the Results section that exposure status accounted for a substantial proportion of explained variance relative to demographic covariates, highlighting its comparative contribution within the multivariable models.**
- **Revision:**
  - **Visual Confirmation:** We added a **Q-Q plot** as Supplementary Material to visually confirm the residual distribution, providing robust evidence for the "approximately normal" assumption despite formal test deviations.
  - **Clinical Magnitude:** We added a paragraph interpreting the clinical magnitude of **Cohen’s d (1.21 for FEV1_z and 1.02 for FVC_z).** We noted that a reduction of **>1 SD represents a significant shift in population lung function percentiles**, **moving an average individual from approximately the 50th percentile to near the 15th percentile**. This demonstrates that the observed differences are **not only statistically significant but clinically meaningful** at the population level.
  - **Model Stability (Sensitivity Analysis):** We clarified and expanded the reporting of regression sensitivity diagnostics presented in Table 6. Specifically, we explicitly interpreted **Cook’s Distance (max < 0.15)** and **Mahalanobis Distance value** to demonstrate that the model estimates are stable and not biased by influential outliers. **Mahalanobis distance values were examined to detect potential multivariate outliers, and no observations exceeded conventional influence thresholds, indicating that the regression estimates were not driven by extreme cases.** This strengthened the justification of model robustness within the manuscript text. **(Please see Revised Manuscript, pages 18-22, lines 579-640 and lines 669-675).**

**6.	Precision Public Health (PPH) Narrative**

- **Reviewer Comment: PPH narrative is repetitive.**
- **Response: We appreciate this observation and agree that the Precision Public Health (PPH) framing required refinement to avoid repetition and promotional tone. We have streamlined the narrative to ensure that PPH is presented as a contextual framework rather than a central rhetorical theme.**
- **Revision: We reduced the mentions of PPH by approximately 50%, and consolidated the conceptual framing into a single, sharp paragraph in the Discussion section. Redundant references across the manuscript were streamlined to improve clarity, balance, and scholarly tone.**

**7.	External Validity (Generalizability)**

- **Reviewer Comment: Findings may not apply to smokers, obese, or multi-morbid populations.**
- **Response: We agree. The "ultrapure" nature of our cohort is a strength for internal validity but a trade-off for generalizability.**
- **Revision: We have added a statement in the Limitations section explicitly clarifying that these findings should not be generalized to smokers, obese individuals, or elderly populations with significant comorbidities, in whom physiological responses to chronic PM₂.₅ exposure may differ. (See page 24, lines 756-763).**

**8.	Structural Revisions (Abstract, Discussion, Conclusion)**

•	**Reviewer Comment:** Abstract and Discussion require tone moderation and reduced redundancy.

•	**Response:** We agree and have carefully revised the relevant sections to moderate tone, reduce redundancy, and improve clarity and conciseness.

•	**Revision:**
– Abstract: Global-first language and strong causal phrasing have been moderated.
– Discussion: Redundant RCT comparisons have been reduced, repeated emphasis on BTPS has been streamlined, and overlapping explanations regarding causal inference have been consolidated to improve clarity and avoid redundancy.
– Conclusion: Reduced by approximately 15% for clarity and focus.

**Response to Reviewer #2 (Dr. Manu Chopra)**

**We thank Dr. Manu Chopra for the positive and constructive evaluation of our work. We have carefully addressed each of the points raised to ensure a more cautious and academically rigorous presentation.**

**1.	Softening Novelty Descriptors**

- **Reviewer Comment: Suggestions to soften terms like “global first,” “pioneering,” and “ultrapure.”**
- **Response: We have followed this suggestion and revised the manuscript to adopt a more cautious tone.**
- **Revision: We removed the terms “global first” and “pioneering.” The phrase has been revised to: *"To our knowledge, few studies in Southeast Asia have applied such a strict decadal residency filter..."* (See page 1, lines 30-32).**

**2.	Sample Size Justification and Power Calculation**

- **Reviewer Comment: Request for a priori power calculation or justification for n=101.**
- **Response: We have strengthened the methodological justification for our sample size by providing both an effect size–based justification and achieved power estimation. Rather than relying solely on conventional post-hoc power testing, we provided an achieved power estimation based on the observed effect sizes to demonstrate that the study was adequately powered to detect clinically meaningful differences. This approach aligns with contemporary methodological recommendations emphasizing effect size magnitude and precision over retrospective hypothesis testing.**
- **Revision:**
  - **Statistical Power:** We have updated the *Statistical Analysis* section to clarify that an a priori detectable effect size calculation informed the study design assumptions, and we subsequently reported an achieved statistical power estimation based on the observed **large effect sizes** (**Cohen’s d ranging from 1.02 to 1.21**). Given these magnitudes, our final cohort of n = 101 achieved statistical power >0.95 at α = 0.01. While the minimum required sample size to detect such large effects was approximately 68 (n = 34 per group), our actual sample size provides an adequate margin to ensure statistical stability.
  - **Model Stability (Sensitivity Analysis):** To further address potential concerns regarding the focused sample size, we have **highlighted the stability diagnostics already provided in Table 6**. Sensitivity analysis using **Cook’s Distance (max < 0.15)** and **Mahalanobis Distance** **(max = 20.338)** confirmed that the regression estimates are robust and not driven by influential outliers. This demonstrates that despite the focused cohort of 101 participants, our findings remain statistically stable and reliable.

**Detailed Justification in Revised Manuscript:**

- **Statistical Power:** See *Statistical Analysis* section on **page 11, lines 376-395**.
- **Stability Diagnostics:** See *Assessment of Model Assumptions* section on **pages 19-21, lines 611-640**.
- **Clinical Meaningfulness:** See *Discussion* section on **pages 21-22, lines 669-675**.

**3.	PM2.5 Exposure Quantification**

- **Reviewer Comment: Clarification on how PM2.5 was quantified (satellite, stations, etc.) and the spatial/temporal resolution.**
- **Response: We have updated the manuscript to provide a more granular description of our two-stage hybrid exposure assessment. We clarified that historical PM₂.₅ information was obtained from government-operated air quality monitoring stations to establish the long-term difference in ambient pollution levels between the two study areas. These publicly available monitoring records report PM₂.₅ concentrations on a daily basis and were used to confirm that Kedoya consistently exhibits substantially higher pollution levels than Pangalengan.**
- **To validate exposure conditions at the residential level during the study period, additional direct field measurements were conducted at three locations within each district using calibrated sensors during 24-hour sampling. We also clarified that participants were recruited from a single administrative sub-district (kelurahan) surrounding the validated measurement sites to minimize within-area exposure variability and strengthen the area-level exposure assignment.**
- **Revision: We have clarified the quantification process as follows:**
  1. **Stage 1 (Historical monitoring data): Publicly available PM₂.₅ monitoring records from government-operated air quality monitoring stations were reviewed to characterize the ambient pollution profile of the two study areas. These monitoring systems report PM₂.₅ concentrations on a daily basis and indicate substantially higher pollution levels in Kedoya compared with Pangalengan in publicly reported monitoring records.**

**2.	Stage 2 (Residential-level validation):** To validate exposure conditions at the neighborhood level during the study period, primary measurements were conducted across three locations in each district using calibrated sensors with 24-hour sampling performed by an independent certified air quality monitoring company.

**3.	Participant assignment:** Participants were exclusively recruited from a single administrative sub-district (kelurahan) surrounding the validated monitoring sites to minimize within-area exposure variability and ensure that the exposure classification reflected neighborhood-level ambient conditions.

**This strategy provided a robust area-level proxy for chronic exposure while reducing the risk of ecological fallacy by ensuring that the exposure contrast was verified at the residential level. (Please see Revised Manuscript, page 13, lines 435-455).**

**4.	Spirometric Terminology**

- **Reviewer Comment: Suggestion to replace “restrictive-type lung function impairment” with more precise terminology.**
- **Response: We agree and have updated the terminology throughout the manuscript.**
- **Revision: We now use the phrase *"pattern consistent with reduced lung volumes without airflow obstruction"* to more accurately reflect the spirometric findings. (See page 17, lines 544-546).**

**5.	Causal Inference Phrasing**

- **Reviewer Comment: Suggestion to change “strengthens causal inference” to “enhances causal inference.”**
- **Response: We have adopted this more conservative phrasing to stay consistent with the observational nature of the study.**
- **Revision: All instances of “strengthened causal inference” have been changed to *"enhanced causal inference."***

We hope these revisions address all concerns and demonstrate the robustness of our study. We look forward to your further assessment. We also acknowledge the reviewer’s decision to make their identity public and appreciate the transparency of the peer review process.

**Best regards,**

Dr. Hari Krismanuel

Universitas Trisakti

[Hari_krismanuel@trisakti.ac.id](mailto:Hari_krismanuel@trisakti.ac.id)
